# Dynamic assessment of the tricuspid annulus in a healthy Asian population: A four‐dimensional echocardiography study

**DOI:** 10.1111/echo.15528

**Published:** 2023-01-17

**Authors:** Jian Wu, Xinyi Huang, Kunhui Huang, Yuan Tian, Qiumei Gao, Biqin Lin, Yiruo Tang, Xu Chen, Maolong Su

**Affiliations:** ^1^ Department of Echocardiography, Xiamen Cardiovascular Hospital of Xiamen University, School of Medicine Xiamen University Xiamen China; ^2^ School of Medicine Xiamen University Xiamen China; ^3^ Xiamen Key Laboratory of Precision Medicine for Cardiovascular Disease Xiamen China; ^4^ Department of Ultrasonography Xiamen Humanity Hospital Fujian Medical University Xiamen China

**Keywords:** Asian population, four‐dimensional echocardiography, reference values, tricuspid annulus

## Abstract

**Background:**

Tricuspid annulus (TA) geometry and function reference values are limited, especially for Asian populations. We aimed to explore TA using four‐dimensional echocardiography (4DE) in a healthy Asian population.

**Methods:**

A total of 355 healthy Asian volunteers (median age 34 years; 52% males) were prospectively enrolled. TA geometry and function were analyzed using 4DE throughout the cardiac cycle.

**Results:**

The TA area, perimeter, and dimensions were smallest at end systole (ES) and largest at late diastole (LD). Normal TA parameters at end diastole (ED) in different sex and age groups were obtained. TA areas, perimeters, and dimensions in males were significantly larger than those in females at ED; BSA‐indexed perimeters and BSA‐indexed dimensions in males were significantly smaller than those in females at ED. TA parameters correlated well with tricuspid valve (TV) tenting, right ventricle (RV), and right atrium (RA) parameters.

**Conclusions:**

Reference values of TA parameters were obtained by 4DE in an Asian population. Quantitative data on TA geometry and function are essential for TA pathology and therapeutics.

## 1 INTRODUCTION

The tricuspid annulus (TA) has a complex three‐dimensional (3D) geometry.^1^, ^2^ Conventional two‐dimensional echocardiography (2DE) cannot comprehensively display the TA in one view, which limits the applicability of geometric assumptions and the reproducibility of 2DE measurements.^3^, ^4^ Nevertheless, to understand TA pathology and therapeutics, it is critical to define the normal geometry and dynamics. Some scholars^1^, ^5^, ^6^ have tried to use three‐dimensional echocardiography (3DE) to evaluate TA during the cardiac cycle. However, to our knowledge, there are no studies of TA nonplanar reference values in a large population of normal Asian adults. Four‐dimensional echocardiography (4DE) can display the complete morphological structure and dynamic changes of the TA and be used to carry out a systematic quantitative analysis of the TA. This study is the first to quantitatively analyze TA in a large cohort of healthy Asian volunteers using transthoracic 4DE.

The purposes of the present study were (i) to obtain accurate reference values for the morphological structure and dynamic changes of the TA in healthy Asian volunteers during the cardiac cycle, (ii) to evaluate the effects of sex and age on TA parameters, and (iii) to evaluate TA parameters in relation to tricuspid valve (TV) tenting, right ventricle (RV), and right atrium (RA) parameters.

## 2 METHODS

### 2.1 Study population

A group of 372 healthy Asian volunteers was prospectively recruited from April 2021 to July 2021. The inclusion criteria were as follows: age ≥18 years, body mass index < 30 kg/m^2^, normal electrocardiogram, normal results on physical examination, no history of cardiovascular or respiratory diseases, and normal results on 2DE (according to the current guidelines and normal values of cardiac size and function of the World Alliance of Societies of Echocardiography Study).^7^, ^8^, ^9^, ^10^, ^11^, ^12^, ^13^

The protocol was approved by the Institutional Ethics Committee, and all volunteers provided written informed consent before undergoing the examinations. Body surface area (BSA) was calculated by using the formula of Du Bois and Du Bois.^14^

### 2.2 Echocardiographic acquisition

Transthoracic 4DE was acquired using a Vivid E95 scanner (GE Vingmed Ultrasound) equipped with a 4 Vc matrix array transducer. Full‐volume, multibeat (3–4 beats) live 4DE images of the TV, RV, and RA was obtained over 3–4 consecutive cardiac cycles from the RV‐focused apical view. The average frame rate was >20 vps. All 4D datasets were stored digitally and analyzed offline.

### 2.3 Echocardiographic measurements

**FIGURE 1:**
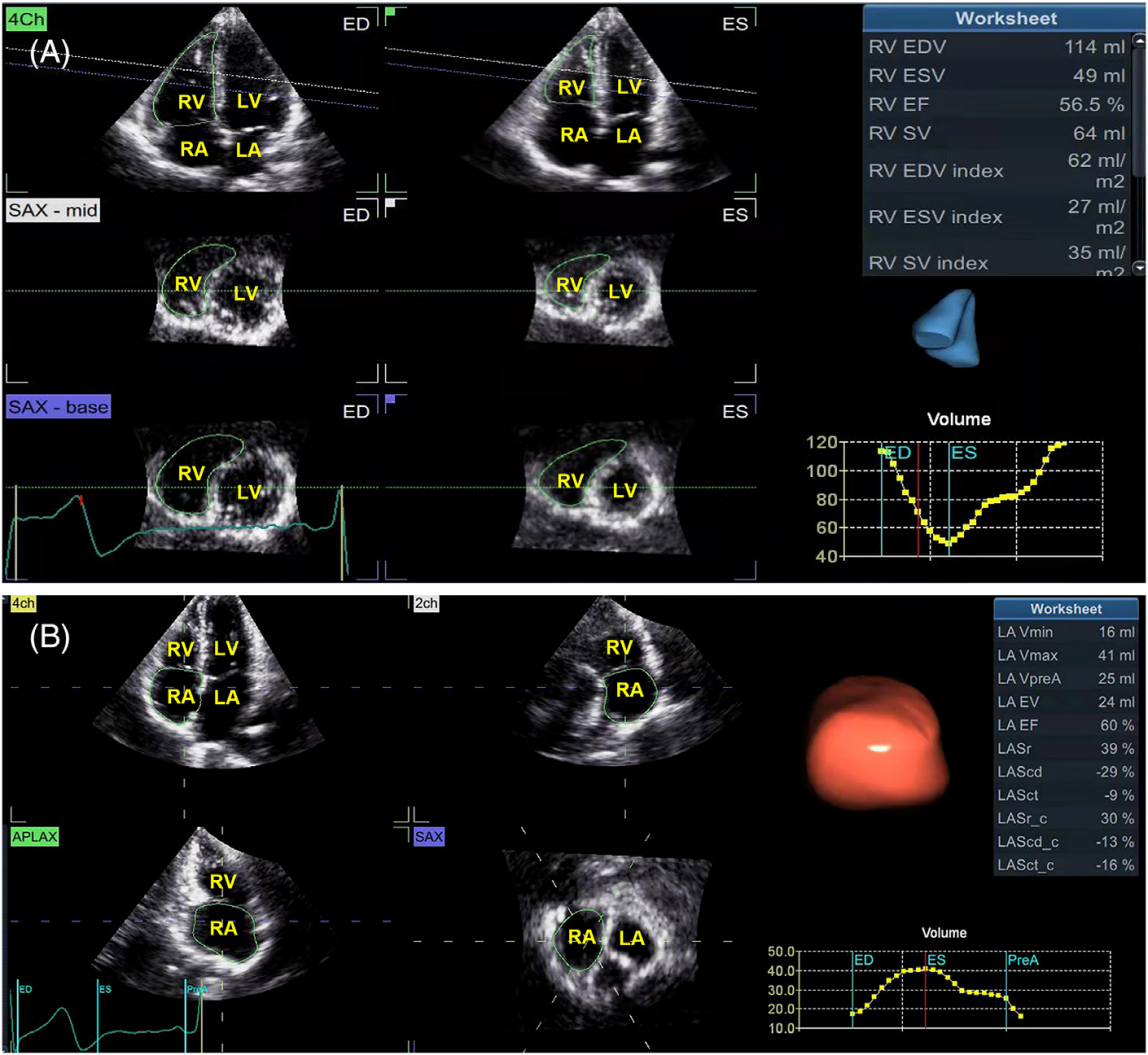
(A) Measurement of right ventricular geometry and function using 4D Auto RVQ; (B) Measurement of right atrial geometry and function using 4D Auto LAQ. ED, end diastole; EDV, end‐diastolic volume; EF, ejection fraction; ES, end systole; ESV, end‐systolic volume; EV, emptying volume; LA, left atrium; LV, left ventricle; RA, right atrium; RV, right ventricle; SV, stroke volume; Vmax, maximal volume; Vmin, minimal volume; VpreA, volume before atrial contraction

4DE images were analyzed using EchoPAC v204 (GE Vingmed Ultrasound). Quantitative parameters of the RV were analyzed with a software package (4D Auto RVQ) (Figure 1A). Quantitative parameters of the RA were analyzed with a software package (4D Auto LAQ) designed for the quantitative analysis of the left atrium (Figure 1B).

**FIGURE 2:**
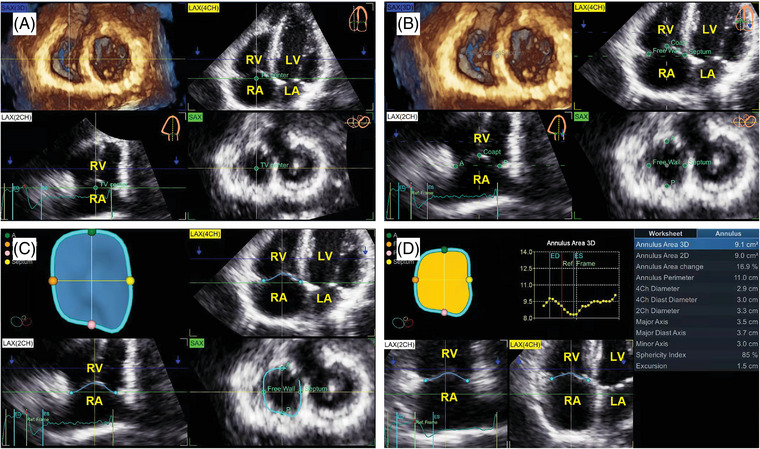
Measurements of tricuspid annulus (TA) and tricuspid valve (TV) geometries and function using 4D Auto TVQ. (A) The aligned views of TA and TV. (B) Manual selections of the anatomical landmarks on the TA and TV. (C) 3D representation and quantitative analysis of TA and TV. (D) Measurements of TA during the cardiac cycle. LA, left atrium; LV, left ventricle; RA, right atrium; RV, right ventricle

**FIGURE 3:**
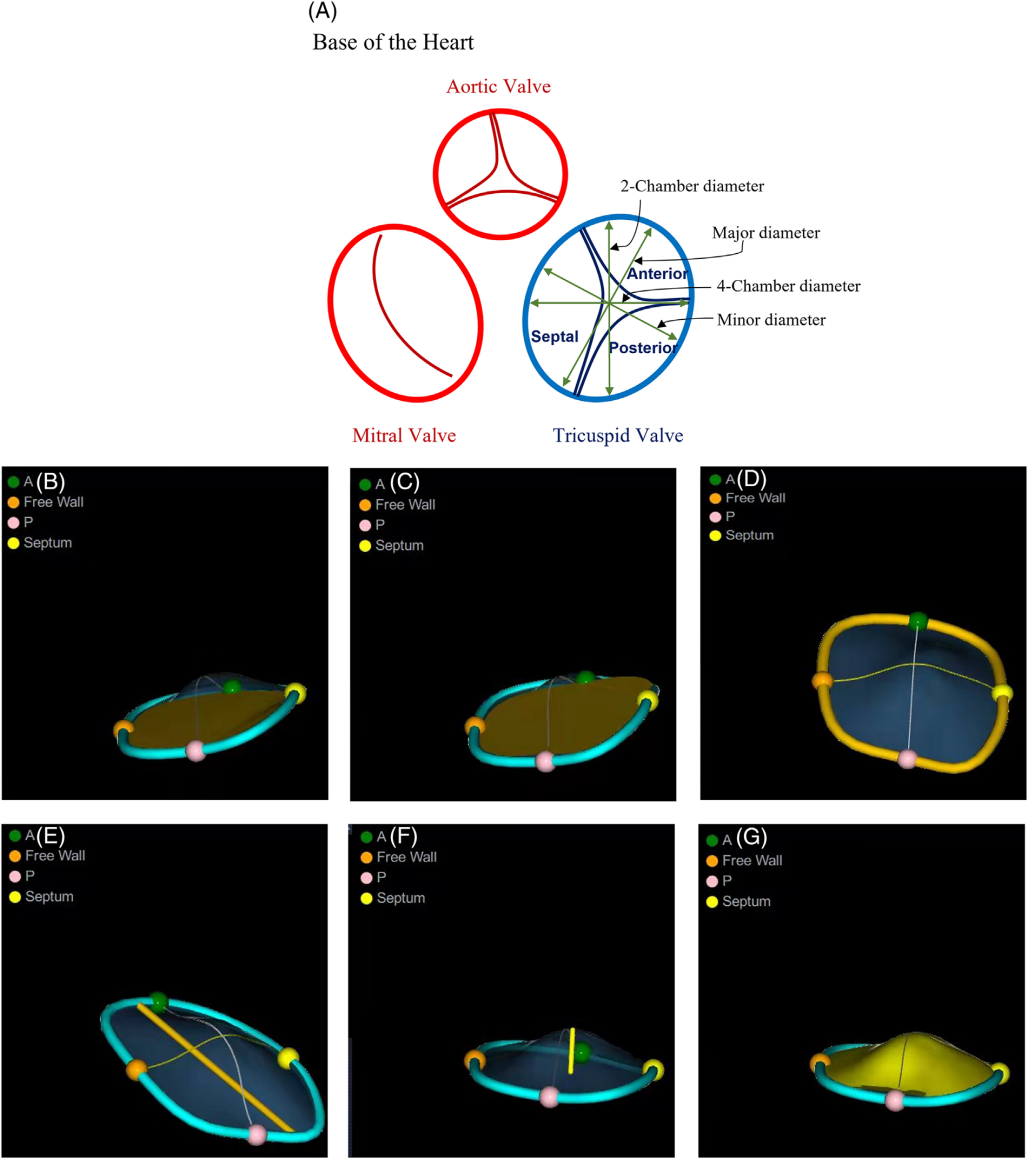
4DE quantitation of tricuspid annulus (TA) and tricuspid valve (TV) geometries. (A) Relative positions of the four‐chamber diameter, the two‐chamber diameter, the major diameter, and the minor diameter in relationship to the TA; (B) TA three‐dimensional area; (C) TA two‐dimensional area; (D) TA perimeter; (E) TA diameter; (F) TV max tenting height; (G) TV tenting volume

Quantitative parameters of the TV were analyzed with a software package (4D Auto TVQ). First, the Align Views stage was used to display a 3D volume rendering to obtain optimal four‐chamber (4Ch) and orthogonal views at the TA level (Figure 2A). Then, the anchor points were placed appropriately (Figure 2B). Segmentation was performed automatically when the final anchor point was set (Figure 2C). Further editing was made on the rotational plane if needed. In the dynamic layout interface, a plot of measurements obtained during the cardiac cycle was displayed with two‐dimensional (2D) slices and a 3D view (Figure 2D). The corresponding dynamic value curve is shown by selecting a measurement in the right panel. Several measurements were calculated automatically from the obtained 4DE images (Figure 3).

### 2.4 Statistical analysis

The normality of variables was assessed with the Kolmogorov–Smirnov test. Normally distributed continuous variables are expressed as the mean ± standard deviation (SD), and those that were not normally distributed were expressed as the median (interquartile range). Normally distributed continuous variables were compared using two‐tailed independent Student's t‐tests or one‐way ANOVA. Nonnormally distributed continuous variables were compared using the Mann–Whitney U test or Kruskal–Wallis test. The Spearman correlation coefficient was used to assess correlations between variables. The 95% confidence interval for normally distributed continuous variables was calculated as the mean ± 1.96 SD. The lowest (2.5%) and highest (97.5%) expected values for nonnormally distributed continuous variables were computed using a bootstrap of 1000 samples.

The intra‐ and inter‐observer variability of the TA parameters at end diastole (ED) was estimated in 20 random subjects by Bland–Altman analysis. One observer analyzed the same 4DE images at two different times to evaluate intra‐observer variability. Two independent, blinded observers analyzed the 4DE images to assess inter‐observer variability.

All data were analyzed using SPSS 25.0 (SPSS, Chicago, IL, USA). When *p* < 0.05, the difference between variables was considered significant.

## 3 RESULTS

Seventeen subjects were excluded from enrollment due to poor 2DE or 4DE images, leading to the feasibility of TA quantitative assessment being 95.4%. The clinical characteristics and right heart parameters of the enrolled population are summarized in Table 1. As expected, the RV and RA ejection fractions were significantly lower, and other right heart parameters were significantly larger in males.

**TABLE 1:** Clinical characteristics and right heart parameters of the enrolled population

	Total (n = 355)	Males (n = 183)	Females (n = 172)	*p*‐value*
Clinical Characteristics				
Age (years)	34 (29‐46)	34 (29‐44)	37 (29‐46)	0.246
BMI (kg/m^2^)	22.7 ± 3.0	23.6 ± 2.6	21.8 ± 3.1	<0.001
BSA (m^2^)	1.68 ± .18	1.81 ± .13	1.54 ± .12	<0.001
RA				
RAV maximum (ml)	40 (35‐46)	44 (40‐50)	36 (33‐40)	<0.001
RAV minimum (ml)	17 (14‐20)	19 (18‐22)	15 (13‐16)	<0.001
RA ejection fraction (%)	57 (55‐60)	56 (54‐58)	60 (57‐61)	<0.001
RV				
RV EDV (ml)	93 (82‐104)	103 (94‐114)	83 (75‐91)	<0.001
RV ESV (ml)	39 (34‐47)	46 (42‐51)	34 (30‐37)	<0.001
RV ejection fraction (%)	57.5 (55.1‐59.9)	55.4 (54.3‐56.4)	59.9 (58.6‐61.0)	<0.001

Abbreviations: BMI, body mass index; BSA, body surface area; EDV, end‐diastolic volume; ESV, end‐systolic volume; RA, right atrium; RAV, right atrial volume; RV, right ventricle; RV Dd base, end‐diastolic right ventricle diameter in 4‐chamber view at basal level; RV Dd mid, end‐diastolic right ventricle diameter in 4‐chamber view at middle level.

^*^ *p*‐value refers to sex differences.

### 3.1 Dynamic changes in the TA during the cardiac cycle

**FIGURE 4:**
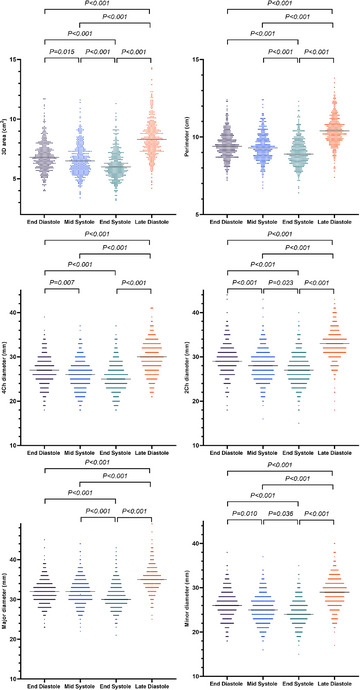
Individual values of tricuspid annulus parameters during the cardiac cycle. Horizontal lines represent median values. 2Ch, two‐chamber; 3D, three‐dimensional; 4Ch, four‐chamber

**FIGURE 5:**
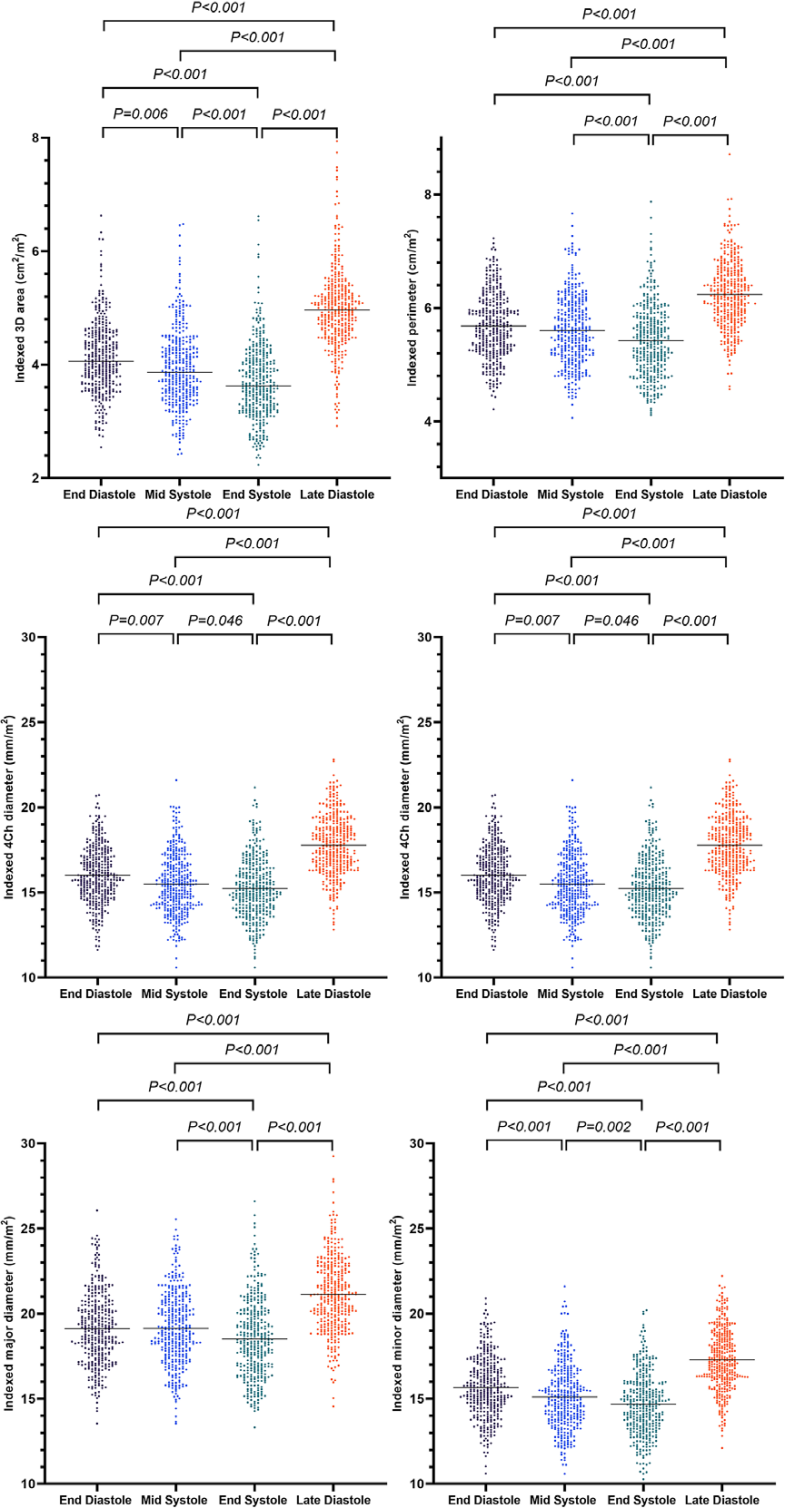
Individual values of BSA‐indexed tricuspid annulus parameters during the cardiac cycle. Horizontal lines represent median values or mean values, appropriately. 2Ch, two‐chamber; 3D, three‐dimensional; 4Ch, four‐chamber; BSA, body surface area

**FIGURE 6:**
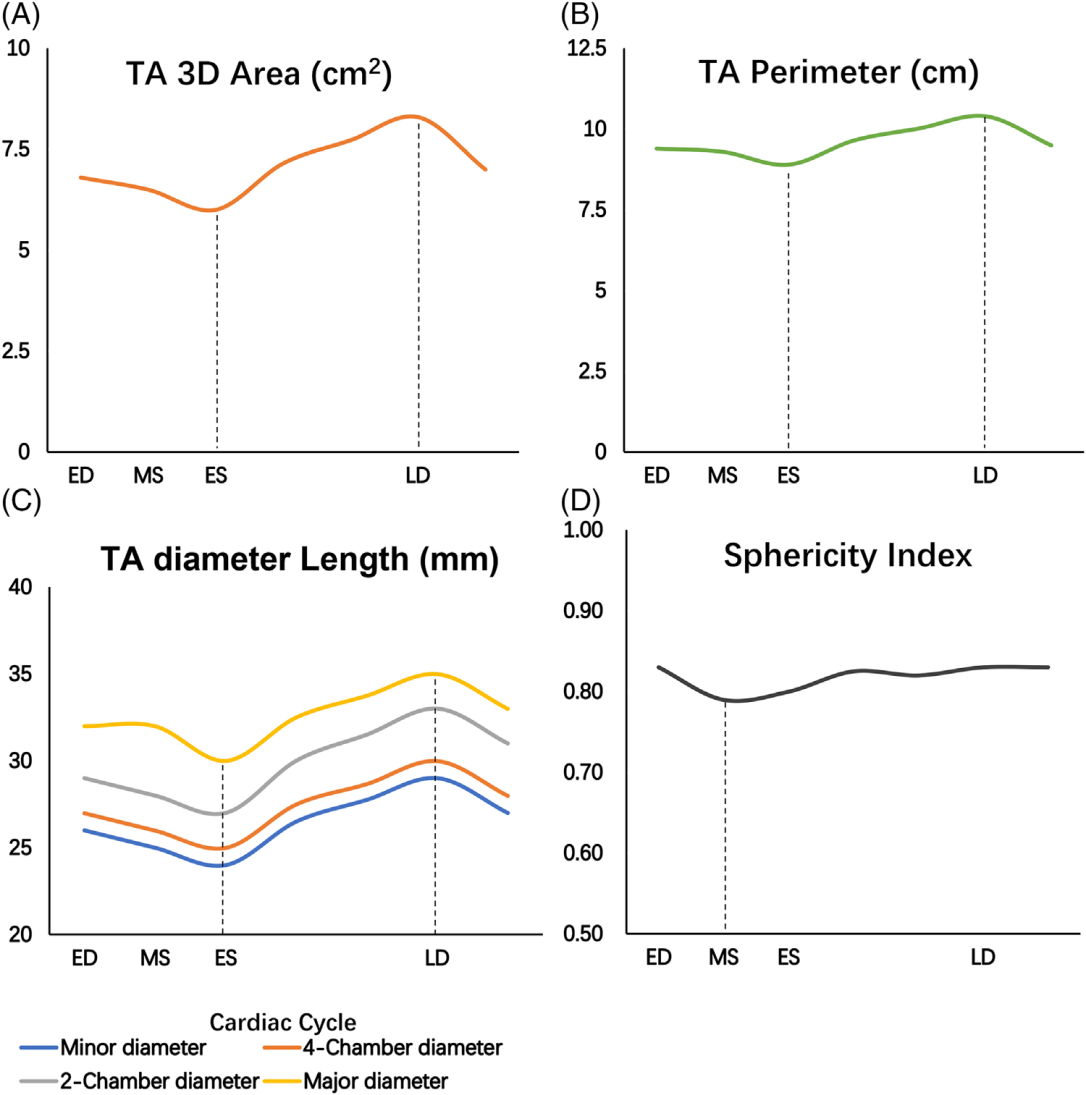
(A) TA 3D area changes during the cardiac cycle; (B) TA perimeter changes during the cardiac cycle; (C) TA diameters changes during the cardiac cycle; (D) TA sphericity index changes during the cardiac cycle. 3D, three‐dimensional; ED, end diastole; ES, end systole; LD, late diastole; MS, mid systole; TA, tricuspid annulus

Table 2, Figures 4 and 5 show the following TA parameters during the cardiac cycle: (1) ED (the first frame just after TV closure); (2) mid systole (MS) (the frame in which the T‐wave began); (3) end systole (ES) (the last frame just before TV opening); and (4) late diastole (LD) (the frame during the PR segment). The nonindexed and BSA‐indexed TA major diameters were significantly longer than the 4Ch diameter and two‐chamber (2Ch) diameter during the cardiac cycle (*p* < 0.001); the nonindexed and BSA‐indexed TA 4Ch diameters were significantly longer than the 2Ch diameter during the cardiac cycle (*p* < 0.001). The 3D area and perimeter of the TA gradually decreased during systole and reached a minimum at ES. Then, they increased gradually, reached a maximum at LD, and then gradually decreased (Figures 6A,B). Between LD and ES, the mean reductions in the TA 3D areas and perimeters were 26 ± 7% and 13 ± 4%, respectively. Figure 6C depicts the changes in the 4DE TA diameters during the cardiac cycle. The TA 3D areas measured at ED, MS, ES, and LD correlated significantly with the 4Ch diameters (r = .677, .673, .684, and .735, respectively), 2Ch diameters (r = .687, .721, .722, and .647, respectively), and major diameters (r = .849, .862, .849, and .827, respectively). The TA sphericity index decreased during systole and increased during diastole (Figure 6D).

**TABLE 2:** Tricuspid annulus parameters obtained using 4DE during the cardiac cycle

	End Diastole	Mid Systole	End Systole	Late Diastole	*p*
**Nonindexed**					
3D area (cm^2^)	6.8 (6.0‐7.6)	6.5 (5.7‐7.3)	6.0 (5.4‐6.9)	8.3 (7.3‐9.2)	^a^, ^b^, ^c^, ^d^, ^e^, ^f^
2D area (cm^2^)	6.6 (5.9‐7.4)	6.3 (5.6‐7.2)	5.9 (5.3‐6.8)	8.1 (7.2‐9.0)	^a^, ^b^, ^c^, ^d^, ^e^, ^f^
Perimeter (cm)	9.4 (8.9‐10.0)	9.3 (8.7‐9.9)	8.9 (8.4‐9.6)	10.4 (9.8‐10.9)	^b^, ^c^, ^d^, ^e^, ^f^
4Ch diameter (mm)	27 (25‐29)	26 (24‐28)	25 (23‐28)	30 (27‐32)	^a^, ^b^, ^c^, ^e^, ^f^
2Ch diameter (mm)	29 (27‐31)	28 (26‐30)	27 (25‐29)	33 (31‐35)	^a^, ^b^, ^c^, ^d^, ^e^, ^f^
Major diameter (mm)	32 (30‐34)	32 (30‐34)	30 (29‐33)	35 (33‐37)	^b^, ^c^, ^d^, ^e^, ^f^
Minor diameter (mm)	26 (24‐28)	25 (23‐27)	24 (23‐26)	29 (27‐31)	^a^, ^b^, ^c^, ^d^, ^e^, ^f^
Sphericity index	.83 (.77‐.89)	.79 (.74‐.85)	.80 (.75‐.86)	.83 (.77‐.88)	^a^, ^b^, ^e^, ^f^
**Indexed (to BSA)**					
3D area (cm^2^/m^2^)	4.1 (3.7‐4.5)	3.9 (3.5‐4.3)	3.6 (3.2‐4.1)	5.0 (4.6‐5.3)	^a^, ^b^, ^c^, ^d^, ^e^, ^f^
2D area (cm^2^/m^2^)	4.0 (3.6‐4.4)	3.7 (3.4‐4.3)	3.6 (3.2‐4.0)	4.9 (4.5‐5.2)	^a^, ^b^, ^c^, ^d^, ^e^, ^f^
Perimeter (cm/m^2^)	5.7 ± .6	5.6 ± .6	5.4 ± .6	6.2 ± .6	^b^, ^c^, ^d^, ^e^, ^f^
4Ch diameter (mm/m^2^)	16.0 ± 1.7	15.5 ± 1.8	15.2 ± 1.8	17.8 ± 1.7	^a^, ^b^, ^c^, ^d^, ^e^, ^f^
2Ch diameter (mm/m^2^)	17.7 ± 2.6	16.9 ± 2.6	16.3 ± 2.6	19.6 ± 2.6	^a^, ^b^, ^c^, ^d^, ^e^, ^f^
Major diameter (mm/m^2^)	19.1 ± 2.1	19.1 ± 2.2	18.5 ± 2.3	21.1 ± 2.2	^b^, ^c^, ^d^, ^e^, ^f^
Minor diameter (mm/m^2^)	15.7 ± 1.8	15.1 ± 1.9	14.7 ± 1.8	17.3 ± 1.8	^a^, ^b^, ^c^, ^d^, ^e^, ^f^

Abbreviations: 2Ch, 2‐chamber; 2D, 2‐dimensional; 3D, 3‐dimensional; 4Ch, 4‐chamber; BSA, body surface area.

^a^ Significant difference between End Diastole and Mid Systole.

^b^ Significant difference between End Diastole and End Systole.

^c^ Significant difference between End Diastole and Late Diastole.

^d^ Significant difference between Mid Systole and End Systole.

^e^ Significant difference between Mid Systole and Late Diastole.

^f^ Significant difference between End Systole and Late Diastole.

### 3.2 Sex differences in TA parameters

**FIGURE 7:**
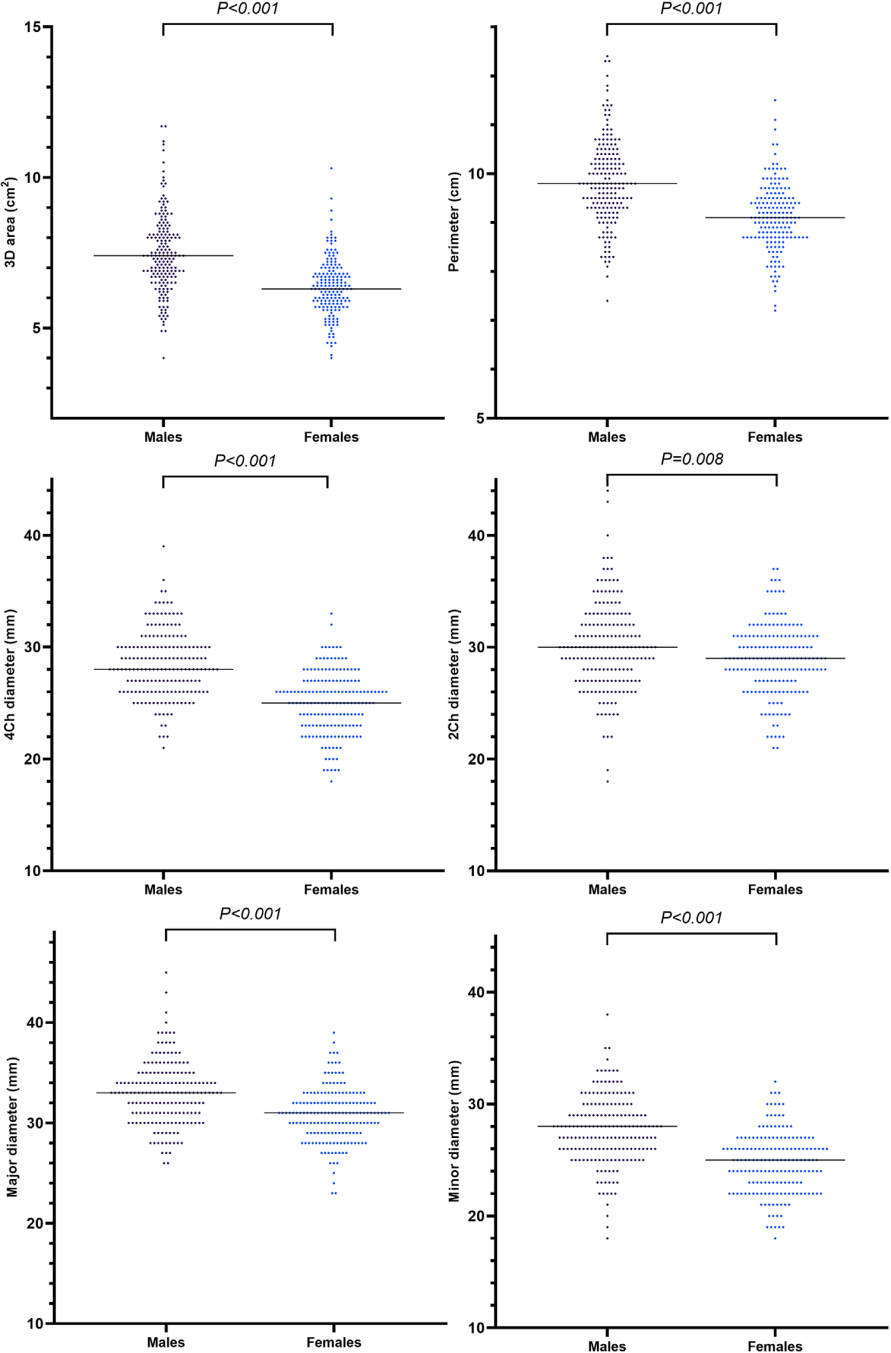
Individual values of tricuspid annulus geometry parameters at end diastole in different sex groups. Horizontal lines represent median values. 2Ch, two‐chamber; 3D, three‐dimensional; 4Ch, four‐chamber

**FIGURE 8:**
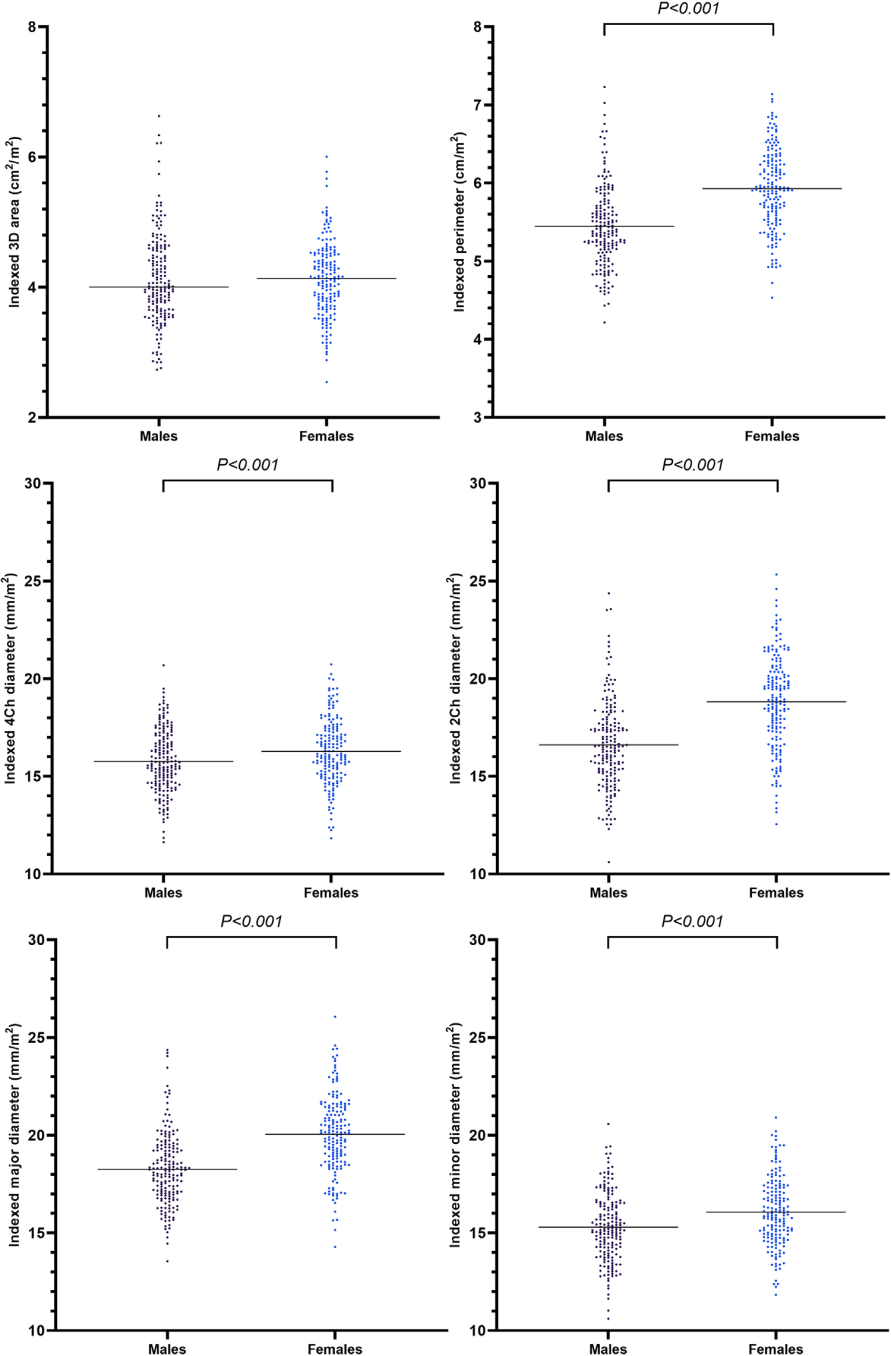
Individual values of BSA‐indexed tricuspid annulus geometry parameters at end diastole in different sex groups. Horizontal lines represent median values or mean values, appropriately. 2Ch, two‐chamber; 3D, three‐dimensional; 4Ch, four‐chamber; BSA, body surface area

TA parameters were compared between sexes at ED (Table 3, Figures 7, and 8). The 3D areas, 2D areas, perimeters, 4Ch diameters, 2Ch diameters major diameters, minor diameters, and sphericity indexes were larger in males at ED. When indexed to BSA, except for the indexed 3D areas and indexed 2D areas at ED, there was no significant difference between the sexes. The indexed 4Ch diameters, indexed 2Ch diameters, indexed major diameters, and indexed minor diameters were smaller in males at ED.

**TABLE 3:** Tricuspid annulus geometry parameters at end diastole in different sex groups

	Males, mean ± SD or median (IQR)	Males, 95% CI or limits of normality ± SE^a^, ^b^	Females, mean ± SD or median (IQR)	Females, 95% CI or limits of normality ± SE^a^, ^b^	*p*‐value*
**Nonindexed**					
3D area (cm^2^)	7.4 (6.7‐8.1)	5.2 ± .1^a^–10.9 ± .4^b^	6.3 (5.8‐6.8)	4.5 ± .2^a^–8.3 ± .2^b^	<0.001
2D area (cm^2^)	7.2 (6.5‐8.0)	5.2 ± .1^a^–10.8 ± .4^b^	6.2 (5.6‐6.7)	4.4 ± .1^a^–8.2 ± .2^b^	<0.001
Perimeter (cm)	9.8 (9.3‐10.4)	8.2 ± .1^a^–11.8 ± .2^b^	9.1 (8.7‐9.5)	7.7 ± .1^a^–10.6 ± .2^b^	<0.001
4Ch diameter (mm)	28 (26‐30)	23.0 ± .6^a^–34.8 ± .5^b^	25 (23‐27)	19.0 ± .7^a^–30.0 ± .5^b^	<0.001
2Ch diameter (mm)	30 (27‐32)	22.0 ± .7^a^–38.0 ± .8^b^	29 (27‐31)	22.0 ± .8^a^–36.0 ± 1.0^b^	0.008
Major diameter (mm)	33 (31‐35)	27.0 ± .3^a^–39.0 ± .6^b^	31 (29‐32)	25.0 ± .6^a^–37.0 ± .6^b^	<0.001
Minor diameter (mm)	28 (26‐29)	22.0 ± .6^a^–33.0 ± .5^b^	25 (23‐27)	19.0 ± .6^a^–30.3 ± .6^b^	<0.001
Sphericity index	85 (78‐90)	66.0 ± 1.7^a^–97.0 ± .5^b^	81 (76‐87)	59.0 ± 2.3^a^–96.0 ± .8^b^	0.001
**Indexed (to BSA)**					
3D area (cm^2^/m^2^)	4.0 (3.6‐4.6)	2.9 ± .1^a^–5.9 ± .2^b^	4.1 (3.7‐4.5)	3.0 ± .1^a^–5.3 ± .1^b^	0.607
2D area (cm^2^/m^2^)	3.9 (3.6‐4.5)	2.8 ± .1^a^–5.8 ± .2^b^	4.0 (3.6‐4.4)	2.9 ± .1^a^–5.3 ± .1^b^	0.857
Perimeter (cm/m^2^)	5.4 ± .5	4.4‐6.4	5.9 ± .5	4.9‐6.9	<0.001
4Ch diameter (mm/m^2^)	15.8 ± 1.6	12.7‐18.9	16.3 ± 1.7	13.0‐19.6	<0.001
2Ch diameter (mm/m^2^)	16.6 ± 2.3	12.1‐21.1	18.8 ± 2.4	14.1‐23.5	<0.001
Major diameter (mm/m^2^)	18.3 ± 1.8	14.8‐21.8	20.1 ± 2.0	16.2‐24.0	<0.001
Minor diameter (mm/m^2^)	15.3 ± 1.7	12.0‐18.6	16.1 ± 1.8	12.6‐19.6	<0.001

Abbreviations: 2Ch, 2‐chamber; 2D, 2‐dimensional; 3D, 3‐dimensional; 4Ch, 4‐chamber; BSA, body surface area; CI, confidence interval; IQR, interquartile range; SD, standard deviation; SE, standard error.

^a^ Lowest expected value.

^b^ Highest expected value.

^*^ *p*‐value differences between sexes.

Table 4 summarizes the fractional changes in the TA parameters between the sexes between LD and ES. Fractional changes in 3D area, 2D area, perimeter, 4Ch diameters, 2Ch diameters, and minor diameters were significantly smaller in females than in males. Fractional changes in the major diameters were not different between the sexes.

**TABLE 4:** Fractional changes of tricuspid annulus parameters between late diastole and end systole in different sex groups

	Fractional Change (%)			
	Total (n = 355)	Males (n = 183)	Females (n = 172)	*p*‐value*
3D area	26 ± 7	27 ± 7	24 ± 7	<0.001
2D area	26 ± 7	27 ± 7	24 ± 8	<0.001
Perimeter	13 ± 4	14 ± 4	12 ± 5	<0.001
4Ch diameter	14 ± 7	15 ± 6	13 ± 8	0.031
2Ch diameter	17 ± 7	18 ± 7	15 ± 7	<0.001
Major diameter	12 ± 6	13 ± 6	12 ± 6	0.087
Minor diameter	15 ± 7	17 ± 6	13 ± 8	<0.001

Abbreviations: 2Ch, 2‐chamber; 2D, 2‐dimensional; 3D, 3‐dimensional; 4Ch, 4‐chamber.

^*^ *p*‐value differences between sexes.

### 3.3 Age differences in TA parameters

**FIGURE 9:**
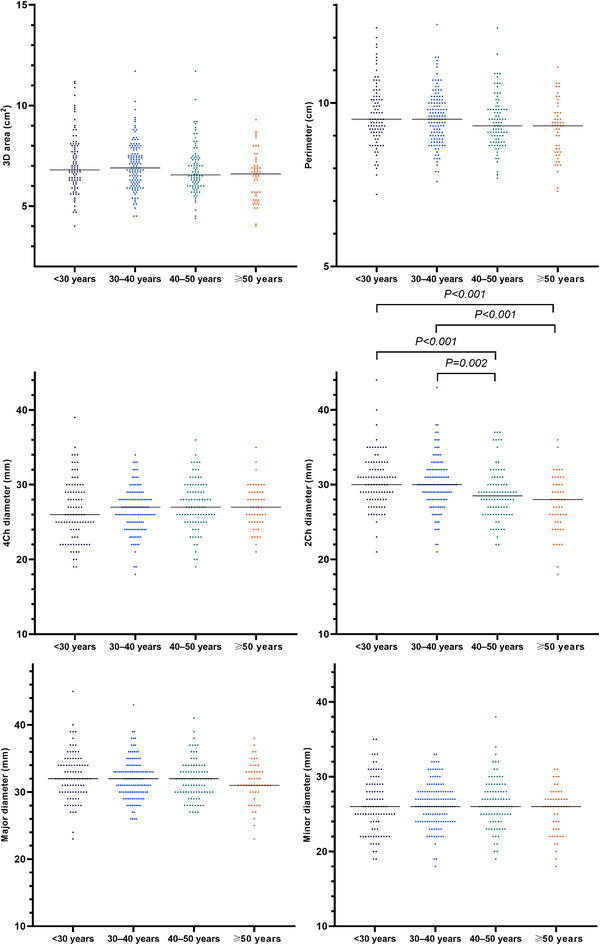
Individual values of tricuspid annulus geometry parameters at end diastole in different age groups. Horizontal lines represent median values. 2Ch, two‐chamber; 3D, three‐dimensional; 4Ch, four‐chamber

**FIGURE 10:**
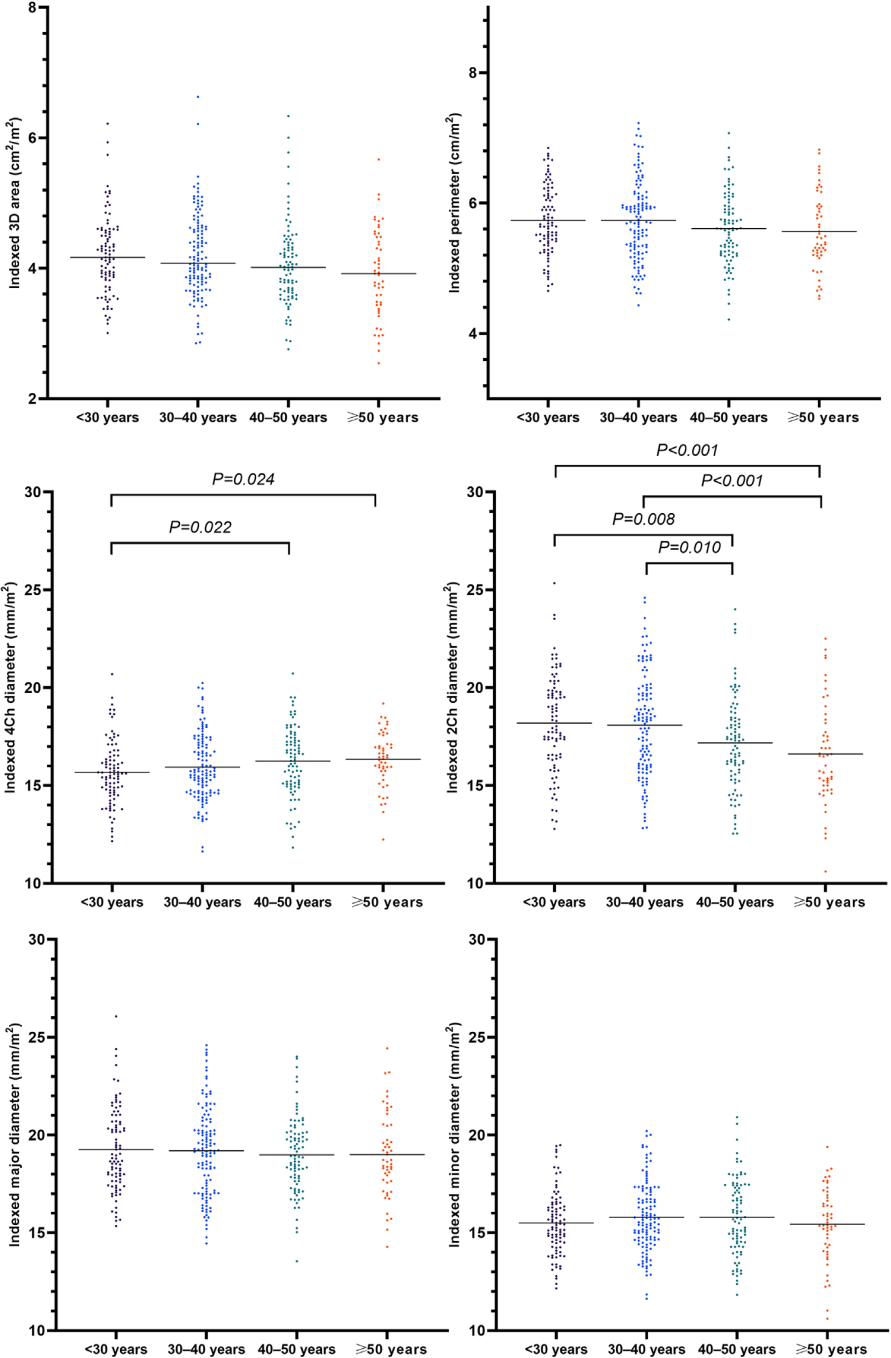
Individual values of BSA‐indexed tricuspid annulus geometry parameters at end diastole in different age groups. Horizontal lines represent median values or mean values, appropriately. 2Ch, two‐chamber; 3D, three‐dimensional; 4Ch, four‐chamber; BSA, body surface area

There was no significant difference in most TA geometry parameters by age group at ED, except for some 2Ch and 4Ch diameters (Table 5, Figures 9 and 10). Nonindexed and BSA‐indexed 2Ch diameters in the <30 and 30–40 years groups were significantly greater than those in the 40–50 and ≥50 years groups. The BSA‐indexed 4Ch diameters in the <30 years group were significantly smaller than those in the 40–50 and ≥50 years groups. Table 6 summarizes the fractional changes in TA parameters and fractional changes in different age groups between LD and ES.

**TABLE 5:** Tricuspid annulus geometry parameters at end diastole in different age groups

	<30 years (n = 91)	30–40 years (n = 125)	40–50 years (n = 88)	≥50 years (n = 51)	*p*
**Nonindexed**					
3D area (cm^2^)	6.8 (6.2‐8.0)	6.9 (6.2‐7.7)	6.6 (6.0‐7.4)	6.6 (5.5‐7.2)	
2D area (cm^2^)	6.7 (5.9‐7.8)	6.7 (6.0‐7.5)	6.4 (5.9‐7.2)	6.5 (5.5‐7.0)	
Perimeter (cm)	9.5 (9.1‐10.2)	9.5 (9.0‐10.0)	9.3 (8.8‐9.8)	9.3 (8.5‐9.7)	
4Ch diameter (mm)	26 (23‐29)	27 (25‐28)	27 (25‐30)	27 (25‐29)	
2Ch diameter (mm)	30 (28‐33)	30 (28‐32)	29 (26‐30)	28 (25‐30)	^a^, ^b^, ^c^, ^d^
Major diameter (mm)	32 (30‐34)	32 (30‐34)	32 (30‐34)	31 (30‐33)	
Minor diameter (mm)	26 (23‐29)	26 (24‐28)	26 (25‐29)	26 (23‐27)	
**Indexed (to BSA)**					
3D area (cm^2^/m^2^)	4.2 (3.8‐4.6)	4.1 (3.8‐4.6)	4.0 (3.6‐4.4)	3.9 (3.4‐4.5)	
2D area (cm^2^/m^2^)	4.1 (3.7‐4.4)	4.0 (3.6‐4.5)	3.9 (3.6‐4.3)	3.8 (3.3‐4.4)	
Perimeter (cm/m^2^)	5.7 ± .5	5.7 ± .6	5.6 ± .6	5.6 ± .6	
4Ch diameter (mm/m^2^)	15.7 ± 1.6	15.9 ± 1.7	16.3 ± 1.8	16.3 ± 1.4	^a^, ^d^
2Ch diameter (mm/m^2^)	18.2 ± 2.5	18.1 ± 2.6	17.2 ± 2.4	16.6 ± 2.6	^a^, ^b^, ^c^, ^d^
Major diameter (mm/m^2^)	19.3 ± 2.1	19.2 ± 2.2	19.0 ± 1.9	19.0 ± 2.2	
Minor diameter (mm/m^2^)	15.5 ± 1.6	15.8 ± 1.8	15.8 ± 1.9	15.4 ± 1.9	

Abbreviations: 2Ch, 2‐chamber; 2D, 2‐dimensional; 3D, 3‐dimensional; 4Ch, 4‐chamber; BSA, body surface area.

^a^ Significant difference between <30 years of age and 40–50 years of age.

^b^ Significant difference between 30–40 years of age and 40–50 years of age.

^c^ Significant difference between 30–40 years of age and ≥50 years of age.

^d^ Significant difference between <30 years of age and ≥50 years of age.

**TABLE 6:** Fractional changes of tricuspid annulus parameters between late diastole and end systole in different age groups

	Fractional Change (%)				
	<30 years (n = 91)	30–40 years (n = 125)	40–50 years (n = 88)	≥50 years (n = 51)	*p*
3D area	25 ± 7	26 ± 7	25 ± 7	28 ± 8	^a^, ^c^
2D area	25 ± 7	26 ± 7	25 ± 7	28 ± 8	^a^, ^b^, ^c^
Perimeter	13 ± 4	13 ± 4	13 ± 4	14 ± 5	^a^
4Ch diameter	14 ± 6	14 ± 7	14 ± 8	16 ± 6	^b^, ^c^
2Ch diameter	16 ± 7	17 ± 6	16 ± 7	17 ± 8	
Major diameter	13 ± 6	13 ± 6	11 ± 6	12 ± 7	
Minor diameter	14 ± 7	14 ± 7	15 ± 8	18 ± 7	^a^, ^b^

Abbreviations: 2Ch, 2‐chamber; 2D, 2‐dimensional; 3D, 3‐dimensional; 4Ch, 4‐chamber.

^a^ Significant difference between <30 years of age and ≥50 years of age.

^b^ Significant difference between 30–40 years of age and ≥50 years of age.

^c^ Significant difference between 40–50 years of age and ≥50 years of age.

### 3.4 Correlations with TV tenting volume and right heart parameters

The maximum TV tenting heights were 8 mm (7–9), 5 mm (4–6), and 4 mm (4–5) at ED, MS, and ES, respectively. The TV tenting volumes were 1.5 ml (1.2–2.0), 1.0 ml (.8–1.2), and .8 ml (.5–1.0) at ED, MS, and ES, respectively. The relationships between the parameters of TA at ED and the parameters of the TV at ED and right heart are detailed in Table 7.

**TABLE 7:** Relationship between parameters of tricuspid annular at end diastole and parameters of tricuspid valve at end diastole and right heart

R	Tricuspid valve tenting height	Tricuspid valve tenting volume	RV EDV	RV ESV	RAV maximum	RAV minimum
3D area	.408*	.790*	.860*	.803*	.929*	.860*
2D area	.402*	.792*	.863*	.808*	.927*	.862*
Perimeter	.393*	.774*	.840*	.780*	.918*	.845*
4Ch diameter	.299*	.553*	.720*	.707*	.705*	.688*
2Ch diameter	.303*	.587*	.470*	.418*	.564*	.491*
Major diameter	.352*	.671*	.705*	.658*	.770*	.699*
Minor diameter	.308*	.621*	.764*	.721*	.783*	.743*

Abbreviations as in Tables 1 and 2.

* *p*<0.05.

### 3.5 Inter‐observer and intra‐observer variability

The intra‐ and inter‐observer variability of the 4DE TA parameters at ED are shown in Table 8 and indicated very high intra‐ and inter‐observer reproducibility.

**TABLE 8:** Intra‐ and inter‐observer variability of tricuspid annulus parameters at end diastole

	Intra‐observer variability			inter‐observer variability		
	Bias	95% CI	ICC	Bias	95% CI	ICC
3D area (cm^2^)	.03	−.62 to .68	.99	−.14	−.63 to .35	.99
2D area (cm^2^)	.03	−.59 to .65	.99	−.11	−.56 to .34	.99
Perimeter (cm)	.01	−.44 to .46	.98	−.09	−.43 to .25	.99
4Ch diameter (mm)	−.10	−1.77 to 1.57	.98	−.30	−2.32 to 1.72	.97
2Ch diameter (mm)	.35	−2.14 to 2.84	.97	−.30	−2.60 to 2.00	.94
Major diameter (mm)	−.05	−2.38 to 2.28	.96	−.20	−2.27 to 1.87	.94
Minor diameter (mm)	−.15	−2.19 to 1.89	.97	−.50	−2.46 to 1.46	.97
Sphericity index	.00	−.07 to .06	.90	−.01	−.09 to .07	.90

Abbreviations: 2Ch, 2‐chamber; 2D, 2‐dimensional; 3D, 3‐dimensional; 4Ch, 4‐chamber; ICC, intraclass correlation coefficient.

## 4 DISCUSSION

This study is the first to use 4DE to quantitatively analyze the TA nonplanar geometry and dynamic changes in a healthy Asian population. Due to the complex 3D geometry of the TA, the parameters of the TA derived from 2DE are often not accurate due to angle and transducer limitations.^2^, ^3^ 3DE has been used to reconstruct the anatomical shape of the TA and obtain relatively accurate reference values of the TA in some studies. However, these studies included predominantly European and American populations.^2^, ^5^, ^15^, ^16^, ^17^ There have been few studies systematically investigating TA in a large cohort of normal Asian populations. Our study derived the normative values of TA in the Asian population by 4DE.

In our study, we found the following: (i) 4DE presents the TA as a whole and its changes in the cardiac cycle well; (ii) 4Ch diameters are smaller than 2Ch diameters, and both are smaller than the major diameters measured by 4DE; (iii) TA diameter reference values should be based on sex and BSA index; and (iv) TA parameters correlate well with the TV tenting volume and RV and RA volumes.

### 4.1 Differences during the cardiac cycle

In our study, the TA 3D area, 2D area, circumference, and diameters had minimal values and maximal values at ES and LD, respectively, which is consistent with the study by Addetia et al.^2^ Conversely, our results were different from the TA dynamic changes found by Miglioranza et al.,^3^ who reported that diameters were largest at early diastole and smallest at MS. The TA sphericity index decreased during systole and increased during diastole. Between LD and ES, the fractional changes in TA 3D area, perimeter, and TA diameters were ∼26%, ∼13%, and ∼15%, respectively. The reference values of the TA at ED may be more meaningful since the TA parameters are the largest in the period of TV closure.

Recently, the multicenter World Alliance Societies of Echocardiography study used transthoracic 3DE to derive the dynamic normative values of the mitral valve apparatus, discovering that the mitral annular size of Asian populations was significantly smaller than that of White and Black populations.^18^ It is reasonable to assume that the same finding will occur in the TA. Guta et al.^16^ used the same software to measure end‐diastolic TA parameters in a group of 83 healthy European volunteers. Although there was a significant age difference between our studied population and theirs, our experiments demonstrated that the TA BSA‐indexed perimeter, BSA‐indexed major diameter, and BSA‐indexed minor at different ages at ED were not significantly different. We found that the TA BSA‐indexed perimeters, BSA‐indexed major diameters, and BSA‐indexed minor diameters at ED of Asian individuals were smaller than those of European individuals (5.7 ± .6 cm/m^2^ vs. 6.6 ± .9 cm/m^2^, *p* < 0.001; 19.1 ± 2.1 mm/m^2^ vs. 22.0 ± 3.0 mm/m^2^, *p* < 0.001; and 15.7 ± 1.8 mm/m^2^ vs. 18.0 ± 3.0 mm/m^2^, *p* < 0.001, respectively). This result may be due to different heart sizes in different racial groups.^10^

### 4.2 Sex and age differences

Our study identified sex as a contributing factor to TA geometry and function. Nonindexed TA parameters in females were significantly smaller than those in males. However, BSA‐indexed TA dimensions at ED were significantly larger in females, which is consistent with the study by Addetia et al.^2^ Fractional changes in TA 3D areas, 2D areas, perimeters, 2Ch diameters and minor diameters were larger in males than females, implying that the TA is more dynamic in normal males than in females. This result is contrary to that from the study by Addetia et al.^2^

Most TV parameters did not differ significantly by age group, suggesting that age is not an independent predictor of TA dilatation. Notably, the fractional changes in some TA parameters were significantly greater in persons aged ≥ 50 than in persons aged < 50, implying that TA mobility is greater in older adults.

### 4.3 Clinical implications

As a noninvasive and convenient technique, 4DE enables the quantification of the dynamic changes in the normal TA geometry during the cardiac cycle.

TR is a common tricuspid disease around the world, and functional tricuspid regurgitation (FTR) accounts for more than 80% of all TRs.^19^, ^20^, ^21^ FTR is often secondary to left ventricular disease, atrial fibrillation, or pulmonary hypertension. These conditions usually result in the development of RV dilation and dysfunction, resulting in TA dilatation.^21^ Recent guidelines showed that patients with more than mild secondary TR with a dilated TA (>40 mm or > 21 mm/m^2^) undergoing left‐heart surgery should be considered for TV surgery.^22^, ^23^ However, our study found that the TA size of Asian populations is smaller than that of European populations; thus, the cutoff values of TA for European populations may not suitable for Asian populations. Therefore, the reference values of TA in our study could provide a fundamental scientific theoretical basis and practical significance for TA pathology, especially for Asian populations.

Over the last several decades, the treatment of moderate to severe TR has received increasing attention, and quantitative research on the normal geometry and function of the TA is ongoing.^22^, ^24^, ^25^, ^26^ At present, tricuspid annuloplasty is widely used in the clinical treatment of FTR.^25^ Tricuspid annuloplasty includes ring annuloplasty and suture annuloplasty, and long‐term follow‐up found that ring annuloplasty was better than suture annuloplasty.^24^, ^27^ In recent years, interventional treatment in TV has also increased dramatically.^28^, ^29^ With the development of various types of tricuspid treatments, obtaining quantitative values of the TA is increasingly critical. The reference values of TA could be used to design optimal coaptation and tricuspid annuloplasty rings that matched the normal tricuspid structure and function for Asian populations, contributing to the development of tricuspid valvuloplasty in Asia.^30^

Moreover, research has also shown that TA 4Ch diameters, major diameters, and minor diameters correlated more strongly with right heart parameters than 2Ch diameters, implying that the enlargement of TA 4Ch diameters from 2DE can well represent the dilation of the right heart. It would be of interest to further explore the relationships between TA function and RV and RA function.

### 4.4 Study limitations

Acquiring and quantifying 4DE datasets using a single provider platform may affect the applicability of these reference values to data obtained from other provider platforms. Moreover, the limited availability of cardiac magnetic resonance and ethical considerations prevented us from verifying the validity of the TA measurements in this study. However, the quantitative measurements of the parameters in our study were well described, and prognostic validation was performed.^31^ Finally, although all subjects were asymptomatic on regular exams, the possibility of subclinical cardiovascular or respiratory disease cannot be ruled out, particularly in elderly subjects.

## 5 CONCLUSIONS

This is the first comprehensive study using 4DE to obtain reference values for TA during the cardiac cycle in a large cohort of the normal Asian population. Sex and BSA should be considered to identify the reference values of TA parameters. TA geometry and function by 4DE are increasingly important in understanding TA pathology and therapeutics.
